# Analgesia Nociception Index Monitoring in Management of Perioperative Analgesia in Total Knee Arthroplasty Surgeries with Femoral Nerve Block

**DOI:** 10.3390/medicina61020213

**Published:** 2025-01-25

**Authors:** Şule Altuncu, Keziban Bollucuoğlu, Rahşan Dilek Okyay, Bengü Köksal İncegül, Çağdaş Baytar, Merve Sena Baytar, Özcan Pişkin, Hilal Ayoğlu

**Affiliations:** Department of Anesthesiology and Reanimation, Zonguldak Bülent Ecevit University Medicine Faculty, Zonguldak 67000, Türkiye; sule.621@hotmail.com (Ş.A.); zengindilek@hotmail.com (R.D.O.); bengukoksal@gmail.com (B.K.İ.); cagdasbaytar31@gmail.com (Ç.B.); drmsbaytar@gmail.com (M.S.B.); drozcanp@gmail.com (Ö.P.); periayogluzku@yahoo.com (H.A.)

**Keywords:** analgesia nociception index, femoral nerve block, total knee arthroplasty, remifentanil consumption, opioid

## Abstract

*Background and Objectives*: The aim of our study is to determine the effects of analgesia nociception index (ANI) monitoring on intraoperative opioid consumption, postoperative analgesia, and the recovery unit length of stay in patients with a preoperative femoral nerve block (FNB) undergoing total knee arthroplasty (TKA) surgery under general anesthesia. *Materials and Methods*: Seventy-four patients in the American Society of Anesthesiologists Physical Status (ASA-PS) I-III scheduled for TKA under general anesthesia were included in this study. After FNB, the patients were divided into two groups (control group (*n* = 35)–ANI group (*n* = 35)). After standard anesthesia induction in both groups, maintenance was conducted using sevoflurane and remifentanil infusion with a bispectral index (BIS) between 40 and 60. In the control group, the intraoperative remifentanil infusion dose was adjusted using conventional methods, and in the ANI group, the dose was adjusted using ANI values of 50–70. The duration of operation, duration of surgery, extubation time, tourniquet duration and pressure, and the amount of remifentanil consumed intraoperatively were recorded. *Results*: Intraoperative remifentanil consumption was lower in the ANI group compared to the control group (*p* = 0.001). The time to reach a Modified Aldrete Scale score (MAS) ≥ 9 was shorter in the ANI group (*p* < 0.001). NRS scores in the recovery unit and 4, 8, 12, and 24 h postoperatively were lower in the ANI group compared to the control group (*p* = 0.006, *p* < 0.05). There was a weak significant inverse relationship between the last ANI values measured before extubation and NRS scores in the postoperative recovery unit (r: −0.070–0.079, *p*: 0.698–0.661). No difference was observed between the groups in other data. *Conclusions*: In patients undergoing TKA with FNB under general anesthesia, ANI monitoring decreased the amount of opioids consumed intraoperatively and postoperative pain scores and shortened the length of stay in the recovery unit. We suggest that ANI monitoring in intraoperative analgesia management may be helpful in determining the dose of opioid needed by the patient and individualized analgesia management.

## 1. Introduction

Total knee arthroplasty is frequently performed as an effective treatment method for end-stage knee osteoarthritis [1]. Severe postoperative pain is observed in 50% of patients, which leads to chronic pain in 15–20% of cases [2]. Postoperative pain is the most important obstacle to the recovery of movement and return to activity after total knee arthroplasty (TKA). Therefore, providing effective analgesia is of great importance for avoiding both acute and chronic complications [3].

Peripheral nerve blocks have an important role in postoperative analgesia as a component of multimodal analgesia. A femoral nerve block (FNB) is commonly used for analgesia and anesthesia in hip and knee surgeries because it provides the analgesia of the anterior femur, knee joint, anterior thigh, ankle, and medial foot [4]. Opioids are also widely used for intraoperative and postoperative pain control [5]. The general aim of intraoperative opioid administration is to suppress the sympathetic response caused by nociception based on hemodynamic parameters. The methods used for the measurement of hemodynamic responses are not direct methods and may lead to the under- or overuse of opioids [6]. There is an increasing use of non-invasive methods for objectively determining intraoperative analgesic requirements under general anesthesia, such as pupillometry, the nociception level index, and the analgesia nociception index (ANI) [7].

Changes in the RR wave interval in an electrocardiogram result from the effects of the autonomic nervous system and are defined as heart rate variability [8]. The ANI is a non-invasive tool whose use involves measuring parasympathetic autonomic activity based on high-frequency heart rate variability analysis [9]. High frequency is used to reflect respiratory sinus arrhythmia and efference and constitutes an important component of vagal activity [8]. Changes in heart rate variability between high frequencies are affected by parasympathetic activity, while changes between low frequencies are affected by both sympathetic and parasympathetic activity [10].

The ANI has values between 0 and 100 based on calculations averaged over 64 s, and the signals received every second are shown in a 1-s window. Two parameters are displayed on a monitor: the instantaneous ANI (ANI_i_) shows the average value of measurements in 56 s, and the average ANI (ANI_m_) shows the average value of measurements over 176 s. These indices are used to detect hemodynamic reactivity during nociceptive stimuli [11]. The aim of this study was to determine the effects of ANI monitoring on intraoperative analgesic dose, postoperative analgesia, and recovery unit and hospital stay in patients who underwent TKA under general anesthesia and underwent preoperative FNB.

## 2. Materials and Methods

This prospective, randomized, controlled study was conducted between 1 March 2022 and 31 December 2023, in Zonguldak Bulent Ecevit University Hospital. Permission to perform this study was first obtained from the Local Ethics Committee (protocol no: 2022/04-21; ClinicalTrials.gov identifier: NCT06236035). This study was designed to include 74 patients aged 18–80 years in ASA-PS I–III who were scheduled to have an elective TKA operation under general anesthesia (patients receiving spinal or combined spinal–epidural anesthesia were not included). Patients were excluded if they declined to participate in this study; had central–autonomic nervous system conditions, neuropsychiatric disease, or cardiac arrhythmia; took medications that affect cardiac autonomic regulation or opioids; or had contraindications for FNB or a known allergy to the drugs to be administered. Patients were also excluded if they received medication for hemodynamic stabilization during the operation (atropine, ephedrine, nitroglycerin, noradrenaline, etc.).

The included patients were informed about this study, written consent was obtained, and the patients were divided into two groups using a closed envelope method: control group (*n* = 35) and ANI group (*n* = 35). The patients’ demographic data and hemodynamic parameters were recorded, and routine monitoring was performed. Both groups were taken to the regional block application room, and 1 mg of intravenous (IV) midazolam was administered. All blocks and anesthesia were performed by the same anesthesiologist (Ş.A.).

Patients were placed in a supine position, the thigh was slightly abducted, and the skin was cleaned using an antiseptic solution. The femoral artery was palpated at 1–2 cm distal to the inguinal ligament, a linear ultrasound probe (8–12 MHz; Esaote Mylab 30 Ultrasound, Esaote, Fishers, IN, USA) was placed, and the femoral nerve under the fascia iliaca was visualized. A 22-gauge 80 mm block needle (BRAUN Stimuplex^®^ Ultra 360^®^, Melsungen, Germany) was connected to a nerve stimulator (Stimuplex HNS 11™, B. Braun Medical Inc., Melsungen, Germany) and advanced from lateral to medial with an in-plane technique.

**Figure 1:**
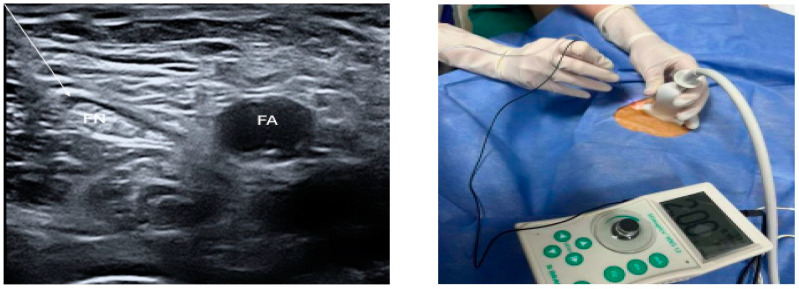
Femoral nerve block using in-plane technique. FN: femoral nerve; FA: femoral artery. Femoral nerve is shown with arrow.

The contraction of the musculus quadriceps femoris (patellar dance) was observed at 0.2–0.5 mA with a frequency of 2 Hz and interval of 0.1 ms. Following negative aspiration, 15 mL of 0.25% bupivacaine was injected, and the local anesthetic distribution was observed (Figure 1). After 20 min, a pinprick test was performed to confirm the block, and the patients were taken to the operating room.

Routine hemodynamic parameter measurements were conducted, and the neuromuscular monitoring of the adductor pollicis muscle was performed with train-of-four (TOF) stimulation (Watch SX Acceleromyograph, Organon Ireland Ltd., Dublin, Ireland). In addition, the BIS (BIS™ Monitoring System, Covidien, Minneapolis, MN, USA) was used to monitor the depth of anesthesia, and the pleth variability index (PVI) was used for fluid monitoring. In the ANI group, electrodes were placed on the dorsal middle and left mid-axillary line facing the heart. Patients were warmed with a forced-air warming blanket throughout the operation.

The standard induction of anesthesia was achieved with 1 mg/kg IV lidocaine (Aritmal^®^ 2%, Osel İlaç, Istanbul, Turkey), 2–3 mg/kg propofol (Propofol-PF^®^ 1%, Polifarma İlaç, Tekirdağ, Turkey), 1 µg/kg fentanyl citrate (Fentanyl-PF^®^, Polifarma İlaç, Tekirdağ, Turkey), and 0.6 mg/kg rocuronium bromide (Muscuron^®^ Koçak Farma İlaçları, Tekirdağ, Turkey). Maintenance was conducted using sevoflurane (Sevorane^®^, Abbvie, Queenborough, UK) and remifentanil infusion (Reksiva^®^, Farma-Tek, Istanbul, Turkey). Mechanical ventilation settings were adjusted according to the following: a respiratory rate of 12 min^−1^; tidal volume of 6–8 mL kg ^−1^; inspiratory-to-expiratory (I:E) ratio of 1:2; positive end-expiratory pressure (PEEP) of 5; fresh gas flow of 4 L min^−1^; O_2_–air ratio of 50:50; and end-tidal CO_2_ at 30–35 mmHg.

Adequate fluid replacement was performed with a PVI value below 14–16%. All patients’ hemodynamic parameters were recorded before induction, after induction, after surgical incision, and every 10 min during the operation. When there were decreases of more than 20% in the baseline heart rate and blood pressure, 0.5 mg of IV atropine and 5 mg of IV ephedrine were administered, respectively, and the patients were excluded from this study.

In the control group, remifentanil infusion was conducted in the range of 0.04–0.3 µg/kg/min when needed according to the change in hemodynamic parameters. During a follow-up, a 20% increase in blood pressure compared to baseline was considered as an indicator of pain, and the remifentanil level was adjusted to 0.04 µg/kg/min; however, if no change was observed in hemodynamic parameters, the infusion dose was increased by 0.04 µg/kg/min at 10 min intervals up to 0.3 µg/kg/min; in the case of a 20% decrease in blood pressure compared to baseline, the dose was reduced to 0.04 µg/kg/min and discontinued. In the ANI group, remifentanil infusion was considered as adequate analgesia if ANI_m_ was between 50 and 70 according to the recorded measurements. If ANI_m_ was below 50, analgesia was considered inadequate, and the remifentanil infusion dose was increased by 0.04 µg/kg min^−1^. When ANI_m_ was >70, the remifentanil dose was decreased by 0.04 µg/kg/min because the patient’s need for analgesia decreased. If the blood pressure remained elevated despite an adequate depth of anesthesia (BIS: 40–60) and analgesia (ANI > 50), tourniquet-induced hypertension was considered to have occurred, 0.1 mg of IV perlinganide was administered, and the patients were excluded from this study. A bolus of 0.1 mg was administered at 3–5 min intervals until blood pressure stabilized.

Patients received 10 mg of IV metoclopramide to prevent postoperative nausea and 1 g of IV paracetamol, 20 mg of tenoxicam, and 1 mg/kg tramadol for multimodal analgesia 30 min before the end of the operation. At the end of surgery, the sevoflurane flow and remifentanil infusion were terminated, and the amount of remifentanil consumed during the operation was recorded. To eliminate neuromuscular blockade, 0.05 mg/kg IV neostigmine and 0.01 mg/kg atropine were administered. When the TOF ratio was 0.9, the patients were extubated and taken to the recovery room.

The time between the beginning and end of anesthesia induction was recorded as the anesthesia duration, the time until the surgical incision and the last suture was recorded as the surgical duration, and the time between the last suture and extubation was recorded as the extubation duration. Hemodynamic parameters and numeric rating scale (NRS) scores were recorded while the patients were in the recovery room, and 0.05 mg/kg IV morphine was administered when the scores were ≥4. In the cases of postoperative nausea and vomiting, 4 mg of IV ondansetron was administered.

The time to reach an MAS score ≥ 9 in the recovery unit; complications (respiratory depression, bronchospasm, airway obstruction, etc.); the NRS score at 4, 8, 12, and 24 h postoperatively (the score was measured by a single person who did not know the patient groups and was not involved in this study); amount of additional analgesic consumption; mobilization time; and hospital stay were recorded. Patients were administered 1 g of IV paracetamol every 6 h postoperatively. As additional rescue analgesics, 75 mg of intramuscular diclofenac sodium was administered if the patient’s NRS score was ≥4, and 1 mg/kg IV pethidine hydrochloride was administered if the NRS score remained ≥4 after 30 min.

### Statistical Analysis

The sample size was analyzed using the program G*Powers 3.1.9.4. In the analysis, total opioid consumption data, which made up our primary outcome in Dundar et al.’s study, were taken as a reference [12]. The minimum number of participants was found to be 27 per group in the sample size analysis, which was performed with 95% confidence intervals and 80% power (control group: 629 ± 422 µg; ANI group: 965 ± 543 µg). A total of 74 patients were included in this study after considering the possibility of 20% loss to follow-up.

Data analysis was performed using the software SPSS 20. Descriptive variables are shown using the mean ± standard deviation, median, and range for quantitative data, as well as frequencies and percentages for qualitative data. The conformity of the data to a normal distribution was evaluated using the Kolmogorov–Smirnov test. Data were analyzed using a *t* test, chi-squared test, Yates-corrected chi-squared test, Fisher’s exact chi-squared test, Mann–Whitney *U* test, and the Spearman and Pearson correlation methods. The analysis results were evaluated at a 95% confidence interval, and *p* < 0.05 was considered significant.

## 3. Results

**Figure 2:**
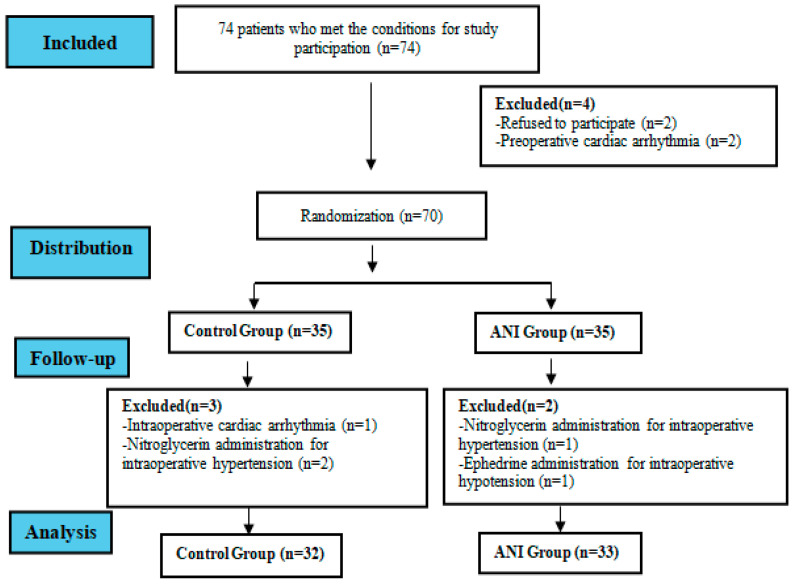
Workflow chart.

There were 74 patients who underwent TKA, but 2 patients were excluded because they declined to participate, and 2 patients were excluded because of preoperative cardiac arrhythmia, leaving 70 patients. In the ANI group, one patient was excluded because of ephedrine treatment, and one patient was excluded because of nitroglycerin treatment. In the control group, 1 patient was excluded because of intraoperative arrhythmia, and 2 patients were excluded because of nitroglycerin treatment, leaving 65 patients who were included in the statistical evaluation (Figure 2). No significant differences were found in the demographic and intraoperative data between the groups (Table 1 and Table 2).

Intraoperative remifentanil consumption was significantly different between groups (Table 3). The recovery unit time; 4-, 8-, 12-, and 24-h postoperative NRS scores, and the time to reach an MAS score ≥ 9 were lower in the ANI group. There was no difference between the groups in terms of morphine consumption in the recovery room and the presence of nausea and vomiting (Table 4). A weak correlation without a statistically significant relationship was found between the last ANI values before extubation and the NRS scores in the recovery unit in the ANI group (Table 5).

## 4. Discussion

This study evaluated the effects of ANI monitoring on intraoperative analgesia management in TKA operations performed under general anesthesia with FNB. The results showed that in the ANI monitoring group, with intraoperative remifentanil consumption, postoperative NRS scores decreased, and the length of stay in the recovery unit was shortened. Although analgesic administration under general anesthesia can be determined by clinical experience (based on the increase in heart rate, blood pressure, and pupil diameter), these methods may lead to the under- or overconsumption of opioids [13,14]. The monitoring of nociceptive–antinociceptive balance under anesthesia and during surgical stimulation is important, so we used the ANI as a non-invasive monitoring method to evaluate pain with objective methods in this study.

Jeanne et al. examined intraoperative ANI monitoring in 27 patients undergoing TKA under general anesthesia and detected early hemodynamic reactivity, its effects on opioid consumption, and hemodynamic changes with 80% sensitivity and 88% specificity [15]. Keeping the ANI value between 50 and 70 leads to ideal analgesia and avoids undesirable hemodynamic effects. A decrease in the ANI_m_ value below 50 has been associated with a hemodynamic response that may occur within 10 min [16]. In the present study, the ANI_m_ and ANI_i_ values were between 50 and 80 in all time periods except at intubation. We believe that this response may be related to the stress response caused by direct laryngoscopy and tracheal intubation despite the provision of an adequate depth of anesthesia. The hemodynamic response can be reduced by increasing the dose of lidocaine or using topical lidocaine.

Recently, the Enhanced Recovery After Surgery (ERAS) protocols were developed to improve postoperative outcomes by implementing multimodal analgesia and approaches that reduce opioid consumption perioperatively [17]. Sabourdin et al. showed that ANI monitoring decreased the remifentanil dose needed but did not change postoperative morphine consumption [18]. Dündar et al. also found that less remifentanil was consumed in the ANI-monitored group compared to the control group [12]. In the present study, the amount of remifentanil consumed was significantly lower in the ANI-monitored group compared to the control group. This result suggests that decreased parasympathetic activity and the early detection of pain development with ANI monitoring reduce intraoperative opioid consumption.

Opioid-induced hyperalgesia (OIH) is a state of sensitivity and paradoxical response to painful stimuli characterized by signal pathway hypersensitivity related to the use of opioid analgesics [19]. The two most important factors in the development of OIH are the dose and duration of infusion. Studies have reported that remifentanil induces OIH and acute opioid tolerance when infused at doses > 0.1 µg/kg min^−1^ [20]. Studies investigating the effects of different doses of intraoperative remifentanil on postoperative opioid consumption have shown that high-dose remifentanil infusion is associated with higher opioid consumption. In these studies, remifentanil infused at a dose higher than 0.2 µg/kg min^−1^ was associated with a lower pain threshold, a larger area of hyperalgesia, and higher pain scores [21,22]. In the present study, anesthesia maintenance was achieved with remifentanil infusion and sevoflurane in both groups. However, we think that the lower postoperative NRS scores in the ANI group were due to the low dose of remifentanil preventing OIH.

Chung et al. applied an FNB and IPACK block (interspace block between the popliteal artery and capsule of the posterior knee) to all patients undergoing TKA under general anesthesia [23]. Remifentanil infusion was used with a target concentration of 2–6 ng/mL with a target-controlled infusion pump in the remifentanil group with intraoperative qNOX (electroencephalogram-based index measuring loss of consciousness and response to nociception) monitoring, while no remifentanil was consumed in the control group. When postoperative opioid consumption was compared, they concluded that postoperative opioid consumption was higher in the remifentanil group, and this was related to OIH.

In the present study, intraoperative remifentanil consumption and postoperative NRS scores were higher in the control group than in the ANI group. We think that the difference in recovery and postoperative NRS scores between the groups is due to the difference in the intraoperative remifentanil doses consumed, which may be related to OIH. Although the postoperative NRS scores measured between the groups showed a difference, we think that there was no difference in the amount of additional analgesics and morphine consumed postoperatively, and the mean NRS scores were ≤4, which did not indicate a clinically significant difference.

A systematic review reported that ANI monitoring predicted postoperative pain and the intraoperative pain level [24]. Boselli et al. found a significant correlation between ANI values measured before extubation and NRS scores in the recovery unit and concluded that the ANI was successful in predicting postoperative pain [25]. Jeanne et al. reported that there was no correlation between intraoperative ANI values and postoperative awake patients’ VAS scores among patients undergoing TKA under general anesthesia [15]. In the present study, we found a weak but significant inverse correlation between ANI values measured before extubation and NRS scores in the recovery unit in the ANI group.

ANI values are affected by not only pain but also the psychological stress and wakefulness of the patient after surgery. Therefore, we believe that the correlation between intraoperative nociception and ANI values may not be equally valid in postoperative awake patients. In addition, since neostigmine and atropine were administered to our patients to reverse neuromuscular blockade at the end of surgery, we think that these drugs may have affected the correlation between ANI values measured before extubation and NRS scores due to their effects on the autonomic nervous system. Additional studies with more participants are needed to clarify this.

Multimodal analgesia provides better analgesia with additive and synergistic effects, as well as a lower side effect profile due to less opioid consumption [26]. In the present study, all patients received FNB, which is a regional technique that reduces the consumption of opioid analgesics in addition to drugs such as paracetamol and NSAIDs in the preoperative period. Özgüner et al. concluded that patients who underwent FNB and adductor canal block (ACB) for TKA under general anesthesia had lower postoperative opioid requirements [27]. They stated that this was due to more sensory nerve branches being affected by FNB because the femoral nerve has intermediate and medial cutaneous branches that innervate the anteromedial aspect of the thigh without entering the adductor canal.

Arzuaga et al. compared the preservation of quadriceps muscle strength, pain control, and functional outcomes in TKA between FNB, femoral triangle block (FTB), and ACB [28]. The quadriceps muscle strength was found to be lower in the FNB group at 6 h postoperatively, while no difference was observed between the other groups at 24 and 48 h. Postoperative pain scores were lower in the FNB group than in the other groups.

They concluded that although FTB and ACB preserved quadriceps muscle strength better than FNB at 6 h postoperatively, there was no difference at 24 and 48 h. Furthermore, FNB was considered better for postoperative pain control, and the resulting muscle weakness did not translate into poor functional results. In the present study, we preferred FNB because it provides effective analgesia, and patients are usually mobilized 24 h after TKA. It is well known that the risk of motor block with FNB increases with the volume and concentration of the drug used. To mitigate this, we employed low doses and diluted drugs. Furthermore, following the orthopedic surgeon’s request, all patients were mobilized after 24 h, and no cases of motor weakness were encountered.

The prolonged use of a tourniquet during general anesthesia may cause an increase in heart rate and blood pressure. This increase may be due to the patient’s pain or the tourniquet-induced hemodynamic response, but it is generally difficult to distinguish these conditions [29]. In tourniquet-induced increases, deepening general anesthesia does not prevent hemodynamic reactions, and antihypertensive drugs may be required. Logier et al. investigated the effects of ANI use on the diagnosis of blood pressure increases that developed during surgical procedures in patients undergoing TKA using a tourniquet under general anesthesia [30]. They concluded that the administration of sufentanil was effective in decreasing blood pressure at low ANI values, but it was insufficient for decreasing blood pressure at high ANI values, and antihypertensive drugs were required. The ANI was found to be effective in determining the etiology of arterial hypertension in tourniquet surgical procedures.

In the present study, high blood pressure despite an adequate depth of anesthesia and analgesia was evaluated as tourniquet-associated hypertension. Although remifentanil infusion was increased according to hemodynamic parameters in the control group and ANI group, if there was no decrease in blood pressure despite the ANI_m_ value being above 50, 0.1 mg of IV nitroglycerin was administered, and the patients were excluded from this study. We think that the ANI may be effective for correct drug selection for etiology in tourniquet surgery, although additional studies are needed in this regard.

This study has some limitations, as follows:

1. We excluded patients with arrhythmia and those taking medications that affect heart rate (beta receptor antagonists, antimuscarinics, vasopressors, perlinganides, and antiepileptic drugs). In addition, we think that the use of neostigmine and atropine to antagonize neuromuscular blockades before extubation may have affected the correlation between ANI values measured before extubation and NRS scores in the recovery unit since they affect the autonomic nervous system.
2. Since we used an opioid with short pharmacokinetics and pharmacodynamics, additional studies are needed to determine the effects of a long-acting opioid.
3. We did not use patient-controlled analgesia devices to monitor postoperative analgesic consumption.

## 5. Conclusions

We determined that ANI monitoring decreased the amount of opioids consumed in intraoperative analgesia management among patients undergoing TKA surgery with preoperative FNB under general anesthesia. This decreased the recovery time and postoperative NRS scores by preventing OIH. Therefore, we believe that ANI monitoring in intraoperative analgesia management may be helpful in determining the dose of opioid needed by the patient and individualized analgesia management.

## Figures and Tables

**Table 1:** Demographic data of patients.

	Control Group (*n* = 32)	ANI Group (*n* = 33)	*p*
**Age**	66.38 ± 8.14 (50–79)	67.91 ± 9.25 (33–80)	0.481
**BMI**	31.28 ± 5.36 (22.6–46.2)	31.53 ± 5.8 (20.4–42.2)	0.857
**Gender (F/M) (*n*)**	29/3	26/7	0.303
**Operated side (R/L) (*n*)**	19/13	16/17	0.528
**ASA-PS (I/II/III) (*n*)**	3/19/10	0/17/16	0.106

Data are expressed as mean ± standard deviation (SD) (min-max). BMI: Body Mass Index; F: Female; M: Male; R: Right; L: Left.

**Table 2:** Intraoperative data of patients.

	Control Group (*n* = 32)	ANI Group (*n* = 33)	*p*
**Duration of Anesthesia (min)**	171.91 ± 18.39 (135–205)	175.12 ± 20.91 (125–213)	0.513
**Surgical Time (min)**	138.53 ± 17.69 (110–180)	139.61 ± 21.13 (100–172)	0.825
**Extubation Time (min)**	9.03 ± 4.52 (3–23)	8.3 ± 4.08 (2–20)	0.498
**Tourniquet Pressure (mmHg)**	305.47 ± 21.3 (280–350)	296.52 ± 18.13 (250–350)	0.072
**Tourniquet Duration (min)**	113.78 ± 15.94 (80–153)	120.88 ± 16.47 (78–145)	0.823

Data are expressed as mean ± standard deviation (SD) (min-max).

**Table 3:** Intraoperative remifentanil consumption of groups.

	Control Group (*n* = 32)	ANI Group (*n* = 33)	*p*
**Remifentanil** **Consumption** **(µg)**	477.5 ± 415.74 (0–1600)	190.91 ± 171.02 (0–550)	**0.001**

Data are expressed as mean ± standard deviation (SD) (min-max).

**Table 4:** Postoperative data of groups.

	Control Group (*n* = 32)	ANI Group (*n* = 33)	*p*
**NRS Score in Recovery Unit**	4 (0–8)	2 (0–7)	**0.006**
**NRS Score T_4_**	3 (1–6)	2 (0–4)	**0.033**
**NRS Score T_8_**	2 (1–5)	2 (0–4)	**0.038**
**NRS Score T_12_**	2 (0–8)	1 (0–3)	**0.007**
**NRS Score T_24_**	2 (0–6)	1 (0–2)	**0.001**
**Morphine Use in** **Recovery Unit +/−**	19/13 (59.4–40.6%)	16/17 (48.4–51.5%)	0.528
**NV in Recovery Unit**	4/28 (12.5–87.5%)	1/32 (3–97%)	0.336
**Time to Reach MAS Score ≥ 9 (min)**	24.97 ± 4.94 (16–36)	18.27 ± 3.43 (13–26)	**0.001**

Data are expressed as mean ± standard deviation (SD) (min-max). NV: nausea–vomiting.

**Table 5:** Comparison of ANI values and recovery NRS scores.

	ANI_i_ Before Extubation	ANI_m_ Before Extubation
**Recovery NRS Scores**	r: −0.070 *p*: 0.698	r: −0.079 *p*: 0.661

r: Pearson correlation coefficient.

## Data Availability

The data supporting the findings of this study are available from the corresponding author upon reasonable request.
